# E‐Learning Attitudes as Predictors of Ethical Practice and Perceived Stress in Virtual Education: A Cross‐Sectional Study From a Resource‐Limited Setting

**DOI:** 10.1002/hsr2.72717

**Published:** 2026-06-28

**Authors:** Mohammad Amin Shadman, Farahnaz Kamali, Razieh Bagherzadeh, Shahnaz Pouladi

**Affiliations:** ^1^ Student Research Committee, Faculty of Nursing and Midwifery Bushehr University of Medical Sciences Bushehr Iran; ^2^ Department of Midwifery, Faculty of Nursing and Midwifery Bushehr University of Medical Sciences Bushehr Iran; ^3^ Department of Nursing, Faculty of Nursing and Midwifery Bushehr University of Medical Sciences Bushehr Iran

**Keywords:** attitude, E‐learning, ethics, stress, students, virtual education

## Abstract

**Background and Aims:**

The rapid growth of virtual education in higher education necessitates an examination of its ethical and psychological implications. This study examines the impact of students' attitudes toward e‐learning on their commitment to ethical behavior and stress levels within virtual learning environments in southern Iran.

**Methods:**

A cross‐sectional, correlational study involved 256 students from Bushehr University of Medical Sciences from October 2022 to March 2023. Participants were selected through quota sampling and completed validated tools, including the Student E‐Learning Attitude Questionnaire, Cohen's Perceived Stress Scale, and a researcher‐designed Ethics in Virtual Education Questionnaire. Data analysis was performed using SPSS v20, with a significance level set at *p* < 0.05.

**Results:**

The mean (±SD) scores were 79.60 ± 11.16 for e‐learning attitudes, 28.04 ± 8.15 for perceived stress, and 149.74 ± 16.87 for ethical practice. Attitudes toward e‐learning significantly predicted ethical practice in virtual education (β = 0.167, 95% CI: 0.077 to 0.429, *p* = 0.005), while no significant relationship was observed between e‐learning attitudes and perceived stress (*p* > 0.05).

**Conclusion:**

Positive attitudes toward e‐learning foster students' adherence to ethical standards in virtual education but do not directly impact perceived stress levels. These findings underscore the importance of fostering constructive perceptions of virtual education and promoting ethical literacy to enhance educational quality and student well‐being.

## Summary

- Virtual education has grown rapidly, raising concerns about students' ethical behavior and psychological well‐being, especially in resource‐limited settings.
- This study reveals that positive attitudes toward e‐learning significantly predict better ethical practices in virtual education, while perceived stress does not directly influence ethical behavior.
- Promoting constructive perceptions of e‐learning and enhancing ethical literacy can improve educational quality and student outcomes in virtual learning environments, particularly in under‐resourced academic contexts.

**Abbreviations**
CVI: Content Validity Index
CVR: Content Validity Ratio
GPA: Grade Point Average

## 1 Introduction

The rapid advancement of communication and information technologies has greatly transformed educational systems worldwide. Among these changes, e‐learning has become a primary method, offering learners increased access, flexibility in time and location, and a wide range of instructional resources [1, 2]. This transition has been especially evident during global crises, such as the COVID‐19 pandemic, during which virtual education played a crucial role in maintaining continuous learning [3, 4]. However, the expansion of virtual learning environments has also introduced new challenges, particularly concerning academic ethics and student well‐being [5, 6].

Ethical behavior in virtual education encompasses a broad spectrum of principles, including academic honesty, respect for privacy, preventing cheating, fulfilling professional duties, and upholding classroom decorum [7, 8]. The remote and often anonymous nature of online learning can complicate the enforcement of these principles, potentially fostering environments where unethical conduct may occur [9]. Evidence indicates that instances of academic dishonesty may be more prevalent in virtual settings than in traditional face‐to‐face classes [8, 9]. Such ethical violations not only undermine the quality of education but can also adversely affect students' mental health and erode trust in institutions.

Simultaneously, perceived stress has become a significant factor impacting students' academic performance and mental health in virtual learning environments. Stress arises when individuals perceive that environmental demands exceed their coping abilities [10]. In e‐learning settings, stressors might include technical problems, reduced peer interaction, unfamiliarity with digital platforms, and heightened academic expectations [6, 11]. Elevated stress levels have been associated with poorer ethical decision‐making and an increased risk of academic misconduct [5].

The potential link between attitudes toward e‐learning and ethical behavior can be grounded in social cognitive theory and models of academic integrity. Social cognitive theory posits that personal factors (such as attitudes and beliefs), environmental factors, and behavior interact reciprocally [12]. In the context of virtual education, a positive attitude toward the e‐learning environment—viewing it as effective, well‐structured, and facilitative—can enhance perceived self‐efficacy and academic engagement, both of which are known antecedents of ethical academic conduct [13, 14]. Conversely, a negative or dismissive attitude may foster psychological distancing from the learning community, potentially reducing perceived accountability and increasing the propensity for misconduct [15]. Furthermore, the Theory of Planned Behavior [16] suggests that attitude toward a behavior is a key determinant of behavioral intention. In this case, a favorable attitude toward the e‐learning system may foster a stronger intention to engage with it ethically, as part of a cohesive ‘academic social contract.’ Thus, examining e‐learning attitudes offers a psychological and contextual lens for predicting adherence to ethical norms in digital learning spaces.

Despite growing global interest in the psychological and ethical aspects of virtual education, research remains limited on the relationship among students' attitudes toward e‐learning, perceived stress levels, and ethical behavior, particularly within Iran's sociocultural context. Most existing studies on the psychological and ethical dimensions of virtual education originate from technologically advanced or well‐resourced academic environments in the Global North, limiting their applicability to under‐resourced regions where digital infrastructure, institutional support, and prior exposure to e‐learning are often constrained [17, 18]. In such resource‐limited settings, factors like inadequate internet connectivity, limited access to digital devices, low digital literacy, and abrupt, unplanned transitions to online learning—as experienced globally during the COVID‐19 pandemic—can profoundly shape students' academic experiences, stress levels, and ethical decision‐making [3, 19]. Furthermore, cultural and contextual norms surrounding education, privacy, and academic integrity may differ significantly, necessitating region‐specific investigations [7].

This study is situated at Bushehr University of Medical Sciences (BPUMS) in southern Iran, a context emblematic of a resource‐limited higher education setting. The university's centralized virtual education unit was established recently, coinciding with the pandemic‐driven shift to remote instruction. Both faculty and students had limited prior familiarity with dedicated e‐learning platforms, relying on the rapid adoption of systems such as Big Blue Button. This context of emerging digital infrastructure, constrained institutional readiness, and compressed adaptation timelines presents a critical case for examining how e‐learning attitudes intersect with perceived stress and ethical practice in virtual environments where support systems are still evolving.

**Figure 1:**
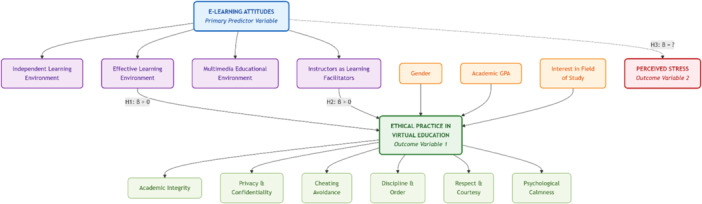
Conceptual framework of the relationships between e‐learning attitudes, perceived stress, ethical practice, and control variables. This model outlines the hypothesized relationships investigated in this cross‐sectional study. E‐learning attitudes (the primary predictor) are conceptualized as comprising four dimensions: independent learning, effective learning, multimedia environment, and instructors as facilitators. These attitudes are hypothesized to directly and positively influence ethical practice (the outcome), which encompasses six dimensions: academic integrity, privacy, cheating avoidance, discipline, respect, and psychological calmness. Perceived stress is included as an exploratory variable to examine its potential association with both attitudes and ethics. The analysis controls for the potential influence of key demographic variables: gender, GPA, and interest in the field of study. Solid arrows represent the primary hypothesized predictive paths. The dashed arrow indicates an exploratory association where the direction of influence was not pre‐specified. The model posits that fostering positive attitudes toward e‐learning may be a key modifiable factor in promoting academic integrity, particularly in resource‐limited settings.

While prior research has often examined either stress or ethics in online learning—typically in stable, well‐resourced contexts—this study addresses two distinct gaps. First, it addresses a theoretical‐conceptual gap by integrating attitudes, perceived stress, and ethical practice within a single predictive model, providing a more holistic understanding of their interrelationships. Second, it addresses a contextual‐practical gap by focusing on a resource‐constrained, non‐Western academic setting in which digital infrastructure and institutional readiness for virtual education are emerging. Specifically, we aim to: (1) assess the levels of e‐learning attitudes, perceived stress, and ethical practice among health sciences students at BPUMS; (2) determine the relationship between e‐learning attitudes and both perceived stress and ethical practice; and (3) identify the extent to which e‐learning attitudes predict ethical practice when controlling for demographic factors. The conceptual framework guiding this study is presented in Figure 1.

The model positions e‐learning attitudes as the primary predictor, comprising four dimensions: (1) independent learning environment, (2) effective learning environment, (3) multimedia educational environment, and (4) instructors as learning facilitators. These attitudes are hypothesized to positively influence ethical practice in virtual education (comprising six dimensions: academic integrity, privacy/confidentiality, cheating avoidance, discipline/order, respect/courtesy, and psychological calmness). The relationship between e‐learning attitudes and perceived stress is exploratory. Control variables (gender, GPA, and interest in field) are included to account for their potential influence on ethical practice. Solid arrows represent hypothesized relationships; dashed arrows indicate exploratory associations or multidimensional constructs.

## 2 Materials and Methods

### 2.1 Study Design and Setting

This study employed a descriptive‐analytical, cross‐sectional approach to examine the relationships among students' attitudes toward e‐learning, perceived stress levels, and ethical practices in virtual education. A correlational design enables researchers to assess multiple variables simultaneously and determine the strength and direction of their associations [20]. The research was carried out at Bushehr University of Medical Sciences, a public institution in southern Iran, from October 2022 to March 2023. The study followed the STROBE guidelines for reporting observational research.

### 2.2 Participants

The target population included undergraduate, master's, and professional doctoral students enrolled in the Faculties of Medicine, Nursing and Midwifery, Allied Health Sciences, Dentistry, and Public Health and Nutrition. Inclusion criteria were: (1) completion of at least one virtual course, (2) access to a smartphone or internet‐enabled device, and (3) willingness to participate. Students were excluded if they were guest (non‐regular) students or did not complete at least one of the study questionnaires.

### 2.3 Sample Size and Sampling Procedure

The required sample size was determined using the rule of thumb for regression analysis, which suggests 10–30 participants per predictor variable. With 11 predictors (10 demographic variables and one primary independent variable), at least 220 participants were needed. To account for a potential 20% dropout rate, the final target sample was set at 264. A quota sampling method was used to ensure proportional representation across academic disciplines and entry years. Within each group, participants were randomly selected via a lottery. If a student declined to participate, a replacement was randomly chosen from the same group.

### 2.4 Data Collection Instruments

Data were gathered through a structured online questionnaire distributed via Google Forms. The tool included four sections:

**Demographic Information Form:** This section collected data on age, gender, marital status, academic level, field of study, semester, interest in the chosen field, grade point average (GPA), prior training in virtual education platforms, number of virtual course credits completed, and the primary software used for virtual learning.

**E‐Learning Attitude Questionnaire:** Adapted from Seyed Naqavi's (2007) validated instrument, this 15‐item scale assessed students' attitudes toward e‐learning across four subscales: (a) independent learning environment, (b) effective learning environment, (c) multimedia educational environment, and (d) instructors as learning facilitators. Items were rated on a 7‐point Likert scale (1 = strongly disagree to 7 = strongly agree), with total scores ranging from 15 to 105. Higher scores indicated more favorable attitudes. The Cronbach's alpha in previous studies ranged from 0.83 to 0.92 [21, 22].

**Perceived Stress Scale (PSS‐14):** Developed by Cohen et al. (1983), this 14‐item scale assesses the extent to which participants perceive their lives as stressful. Items are rated on a 5‐point Likert scale (0 = never to 4 = very often), with reverse scoring applied to seven items. Total scores range from 0 to 56, with higher scores indicating greater perceived stress [10]. The Persian version has shown acceptable reliability (Cronbach's α = 0.80) [23].

**Ethical Practice in Virtual Education Questionnaire:** This researcher‐developed instrument includes 35 items across six subscales: academic integrity, privacy and confidentiality, avoidance of cheating, discipline and order, respect and courtesy, and psychological calmness. Items are rated on a 5‐point Likert scale (1 = never to 5 = always), with reverse scoring for four items. Total scores range from 35 to 175, with higher scores indicating stronger adherence to ethical principles. Psychometric evaluation shows a content validity index (CVI) of 0.93, a content validity ratio (CVR) of 0.84, and a Cronbach's alpha of 0.92 [24].

### 2.5 Data Collection Procedure

After receiving approval from the university's Research Council and Ethics Committee (IR.BPUMS.REC.1401.084), the researcher obtained cooperation letters from the relevant faculties. Class schedules were reviewed in collaboration with faculty administrators, and eligible students were approached during class sessions. The study objectives were explained, and verbal consent was obtained before collecting students' contact information. The questionnaire link was then shared via social messaging platforms, including WhatsApp, Eitaa, and Bale. Participation was voluntary, and informed consent was implied upon submission of the completed questionnaire. Data collection continued over 5 months.

### 2.6 Statistical Analysis

Data analysis was conducted using SPSS version 20 [25] and was informed by our conceptual framework (Figure 1), which specified the hypothesized relationships between predictor, outcome, and control variables. It should be noted that the term ‘predictor’ is used in a statistical sense within the regression model, indicating variables that explain variance in the outcome, and does not imply causal inference due to the cross‐sectional design. The primary, pre‐specified analyses were: (1) the bivariate Pearson correlations between the three main variables, and (2) the hierarchical linear regression model with ethical practice as the outcome. Additional exploratory analyses included examining subscale correlations (Table 4) and univariate associations between all demographic variables and ethical practice (Table 1). Prior to analysis, the normality of the main continuous variables (total scores for e‐learning attitudes, perceived stress, and ethical practice) was assessed and confirmed using visual inspection of histograms and the Kolmogorov‐Smirnov test [26], with all tests confirming normality (*p *> 0.05). This justified the use of parametric tests [27]. Descriptive statistics, including means, standard deviations, and frequencies, were computed. The Pearson correlation coefficient (r) was used to examine the strength and direction of linear relationships between attitudes toward e‐learning, perceived stress, and ethical practice. Hierarchical linear regression models [28] were constructed to identify predictors of ethical practice while controlling for relevant demographic variables (entered in Block 1). Assumptions for regression, including linearity, independence of errors (Durbin‐Watson statistic), homoscedasticity (visual inspection of scatterplots), and absence of multicollinearity (Variance Inflation Factor, VIF < 10), were evaluated and met [26].

**Table 1:** Association between demographic variables and ethical practices in virtual education among student participants (*n* = 256), 2023.

Variable		*N* (%) or Mean*(±SD) ** or Median***	Total application of ethics	
			Standardized regression coefficient	*p* value
Age (years)		22.25* (2.38) **	0.022	0.723
Semester		5***	−0.049	0.439
Gender	Female	155 (60/5%)	Ref	—
	Male	101 (39.5%)	−0.161	0.010
Marital status	Married	18 (7.0%)	Ref	—
	Single	238 (93.0%)	0.038	0.550
Interest in field of study	Low	21 (8.2%)	Ref	—
	Moderate	109 (42.6%)	0.056	0.619
	High	126 (49.2%)	0.337	0.003
Academic level	Professional Doctorate	97 (37.9%)	Ref	—
	Master's Degree	10 (3.9%)	0.040	0.536
	Associate and Bachelor's Degrees	149 (58.2%)	−0.010	0.871
Grade point average (GPA)	GPA > 18	49 (19.1%)	Ref	—
	16–18	147 (57.4%)	−0.154	0.055
	GPA < 16	60 (23.4%)	−0.291	< 0.001
Participation in virtual education software workshops	No	200 (78.1%)	Ref	—
	Yes	56 (21.9%)	−0.025	0.691
Virtual instruction credits	Less than 4 Credits	30 (11.7%)	Ref	—
	More than 4 Credits	226 (88.3%)	0.092	0.143
Field of study	Medicine	78 (30.5%)	Ref	—
	Dentistry	21 (8.2%)	0.031	0.648
	Nursing–Midwifery	52 (20.3%)	0.020	0.781
	Allied Health and Library Sciences	63 (24.6%)	−0.015	0.839
	Public Health and Nutrition	42 (16.4%)	−0.008	0.914
Virtual teaching software	Adobe connects	20 (7.8%)	Ref	—
	Skype	16 (6.3%)	0.013	0.870
	Sky room	26 (10.2%)	−0.141	0.118
	Big blue button	178 (69.5%)	0.041	0.704
	Google Meet or Chrome	16 (6.3%)	−0.004	0.963

*Note:* * The figures provided indicate mean values, ** The figures provided indicate standard deviation values, *** The figures provided indicate median values, *** The median is reported for ‘Semester’ as its distribution was non‐normal. † Abbreviations and statistical symbols used in tables: GPA, Grade Point Average; r, Pearson correlation coefficient; β, standardized regression coefficient; R^2^, coefficient of determination; CI, 95% Confidence Interval; Ref, reference category.

The sample size was determined using a common rule‐of‐thumb for multiple regression, which suggests 10–30 participants per predictor variable [29]. With 11 predictors anticipated, a minimum sample of 220 was required. A target sample of 264 was set to account for potential attrition.

The significance threshold was set a priori at α = 0.05. All reported *p* values are from two‐sided tests. Effect sizes are presented as correlation coefficients (r) or standardized regression coefficients (β), each accompanied by their 95% confidence intervals (CI). All analyses were performed using IBM SPSS Statistics for Windows, Version 20.0 (IBM Corp., Armonk, NY, USA). The reporting of statistical methods and results adheres to the guidelines proposed by Assel et al. for transparent reporting in clinical research [30]. This study follows the STROBE guidelines for reporting observational research [31].

### 2.7 Consent to Participate

All participants were fully informed about the study's purpose, procedures, and voluntary nature before participating. Informed consent was obtained from each participant before data collection. For online questionnaires, consent was explicitly indicated by participants selecting an “I agree to participate” option before accessing the survey. Participants were assured that their participation was entirely voluntary and that they could withdraw from the study at any time without penalty. No personally identifiable information was collected, and all responses were kept strictly confidential.

### 2.8 Ethical Considerations

The Ethics Committee of Bushehr University of Medical Sciences approved the study protocol. Participants were assured of the confidentiality and anonymity of their responses. To minimize social desirability bias, participants were assured of complete anonymity and confidentiality, and the questionnaire did not collect any personally identifiable information. Data were reported in aggregate form and used solely for research purposes.

## 3 Results

### 3.1 Participant Characteristics

A total of 256 students completed the study, yielding a response rate of 97.0% relative to the target sample size of 264. The average age of participants was 22.25 years (SD = 2.38). The majority were female (*n* = 155, 60.5%) and single (*n* = 238, 93.0%). Most participants were enrolled in associate or bachelor's degree programs (*n* = 149, 58.2%), and nearly half (*n* = 126, 49.2%) reported high interest in their field of study. Only 19.1% (*n* = 49) had a GPA above 18, while 23.4% (*n* = 60) had a GPA below 16. Approximately 88.3% (*n* = 226) had completed more than four credits of virtual coursework, and 69.5% (*n* = 178) reported using Big Blue Button as their primary virtual learning platform (Table 1).

### 3.2 Descriptive Statistics of Main Variables

The mean total score for attitudes toward e‐learning was 79.60 (SD = 11.16), indicating generally favorable perceptions. The mean perceived stress score was 28.04 (SD = 8.15), reflecting moderate stress levels. The mean total score for ethical practice in virtual education was 149.74 (SD = 16.87), suggesting a high level of ethical adherence among participants. Subscale means are presented in Table 2.

**Table 2:** Mean and standard deviation of attitudes toward virtual education, perceived stress, and ethical application in virtual instruction among students participating in the 2023 Study (*N* = 256).

Variable	Domain	Mean	SD
Attitude Toward Virtual Education (15–105)	Independent Learning Environment	30.91	5.54
	Effective learning Environment	15.13	3.58
	Multimedia Educational Environment	17.61	2.64
	Instructors as Learning Facilitators	15.96	3.05
	Total Attitude	79.60	11.16
Ethical Application in Virtual Instruction (35–175)	Academic Integrity	13.05	1.84
	Privacy and Confidentiality	13.82	1.63
	Avoidance of Cheating	26.69	6.42
	Discipline and Order	46.71	5.97
	Respect and Courtesy	40.27	4.52
	Psychological Calmness	9.20	1.18
	Total Ethical Application in Virtual Instruction	149.74	16.87
Perceived Stress (0–56)		28.04	8.15

### 3.3 Bivariate Associations

Pearson correlation analysis showed a significant positive relationship between attitudes toward e‐learning and ethical practice in virtual education (r = 0.168, 95% CI: 0.046 to 0.285, *p* = 0.007). No significant relationship was observed between attitudes toward e‐learning and perceived stress (r = −0.011, 95% CI: −0.122 to 0.100, *p* = 0.861), nor between perceived stress and ethical practice (r = 0.034, 95% CI: −0.080 to 0.147, *p* = 0.584) (Table 3).

**Table 3:** Pearson correlation coefficients among main study variables (*N* = 256).

Variable	1. Attitude toward E‐learning	2. Perceived stress	3. Ethical practice
1. Attitude Toward E‐learning			
Correlation coefficient (r)	1		
95% CI	—		
*p*‐value	—		
2. Perceived stress			
Correlation coefficient (r)	−0.011	1	
95% CI	−0.122, 0.100	—	
*p*‐value	0.861	—	
3. Ethical practice			
Correlation coefficient (r)	0.168	0.034	1
95% CI	0.046, 0.285	−0.080, 0.147	—
*p*‐value	0.007	0.584	—

Further analysis of the attitude subscales revealed that perceiving e‐learning as an effective learning environment (r = 0.170, *p* = 0.007) and perceiving instructors as learning facilitators (r = 0.140, *p* = 0.025) were significantly associated with higher ethical practice scores. No other subscales showed statistically significant relationships (Table 4).

**Table 4:** Correlations between E‐Learning attitude subscales, perceived stress, and total ethical practice (*N* = 256).

E‐Learning attitude subscale	Perceived stress	Total ethical practice
Independent Learning Environment		
Correlation Coefficient (r)	−0.043	0.097
95% CI	−0.155, 0.070	−0.026, 0.217
*p*‐value	0.494	0.122
Effective Learning Environment		
Correlation Coefficient (r)	0.114	0.170
95% CI	−0.009, 0.234	0.049, 0.287
*p*‐value	0.068	0.007
Multimedia Educational Environment		
Correlation Coefficient (r)	−0.118	0.115
95% CI	−0.238, 0.004	−0.007, 0.234
*p*‐value	0.060	0.065
Instructors as Learning Facilitators		
Correlation Coefficient (r)	0.005	0.140
95% CI	−0.108, 0.118	0.017, 0.259
*p*‐value	0.939	0.025
Total Attitude Score		
Correlation Coefficient (r)	−0.011	0.168
95% CI	−0.122, 0.100	0.046, 0.285
*p*‐value	0.861	0.007

### 3.4 Associations With Demographic Variables

Univariate regression analysis showed that male gender (β = −0.161, *p* = 0.010) and GPA below 16 (β = −0.291, *p* < 0.001) were significantly linked to lower ethical practice scores. Conversely, a high level of interest in the field of study was positively connected to ethical practice (β = 0.337, *p* = 0.003). Other demographic variables, including age, marital status, academic level, and participation in virtual education workshops, were not significantly related to ethical practice (Table 1).

### 3.5 Multivariate Regression Analysis

Two hierarchical linear regression models were developed to identify predictors of ethical practice in virtual education. Model 1 included only demographic variables and accounted for 12.2% of the variance in ethical practice scores (R^2^ = 0.122, F = 6.928, *p* < 0.001). In Model 2, attitudes toward e‐learning were added, leading to a statistically significant improvement in model fit (R^2^ = 0.149, ΔR^2^ = 0.027, F change = 8.006, *p* = 0.005).

In the final model, attitudes toward e‐learning were a significant positive predictor of ethical practice (β = 0.167, 95% CI: 0.077 to 0.429, *p* = 0.005), indicating that for each one‐point increase in attitude score, the ethical practice score increased by 0.167 points, holding other variables constant. A high interest in the field of study remained a significant positive predictor (β = 0.284, 95% CI: 2.100 to 17.053, *p* = 0.012), while GPA below 16 continued to be a significant negative predictor (β = −0.191, 95% CI: −13.954 to −1.210, *p* = 0.020). Male gender approached significance (β = −0.103, *p* = 0.091) but did not reach the conventional threshold (Table 5).

**Table 5:** Relationship between attitudes toward virtual education and total ethical practice score in virtual education, considering the effects of demographic variables among student participants in the 2023 Study (*N* = 256).

Variable		Model 1		Model 2		
		Standardized regression coefficient	*p* value	Standardized regression coefficient	*p* value	95% Confidence interval (*CI*)
Male Gender (Reference: Female)		−0.106	0.087	−0.103	0.091	−7.653; 0.564
Interest in Field of Study (Reference: Low Interest)	Moderate	0.027	0.811	0.053	0.635	−5.685; 9.305
	High	0.275	0.017	0.284	0.012	2.100; 17.053
Academic GPA (Reference: GPA > 18)	16–18	−0.072	0.367	−0.068	0.387	−7.579; 2.950
	GPA < 16	−0.176	0.033	−0.191	0.020	−13.954; −1.210
Attitude toward Virtual Education		—	—	0.167	0.005	0.077; 0.429
R square		0.122		0.149		
F (*p* value)		6.928 (< 0.001)		7.270 (< 0.001)		
R‐squared change		—		0.027		
F change (*p* value)		—		8.006 (0.005)		

*Note:* The dependent variable: Total Ethical Application in Virtual Education. Because of multicollinearity, the overall score for this variable was included in the regression analysis rather than the specific attitude domains related to virtual education.

## 4 Discussion

The present study extends the existing literature by demonstrating that, in a resource‐limited academic setting, positive attitudes toward e‐learning significantly predict ethical practice, whereas perceived stress is not directly associated with ethical practice. This contrasts with findings from more technologically advanced contexts, where stress has frequently been linked to academic misconduct [32, 33]. The divergence may be attributed to contextual factors such as communal coping strategies, familial support, or differing perceptions of academic pressure in collectivist cultures. Furthermore, unlike prior studies that focused on ethical behavior or stress in isolation, this investigation integrates both constructs within a single model, providing a more comprehensive understanding of student experiences in virtual learning environments. It should be noted that the high self‐reported ethical scores may partially reflect social desirability bias, a common limitation in ethics research. However, the anonymity of responses and the use of validated subscales strengthen the credibility of the findings.

The emphasis on instructor facilitation and perceived effectiveness of e‐learning as key attitudinal drivers of ethics also adds nuance to existing models, which have often emphasized institutional policy over pedagogical perception. The following discussion addresses each of our three research objectives in turn, situates the findings within the broader literature, and draws practical implications for health sciences education in resource‐limited contexts.

### 4.1 Attitudes Toward E‐Learning and Ethical Application

The finding that positive e‐learning attitudes predict ethical practice aligns with theoretical models such as Social Cognitive Theory [12] and the Theory of Planned Behavior [16]. According to these frameworks, attitudes shape behavioral intentions and self‐efficacy. Students who perceive e‐learning as effective and well‐facilitated are likely to experience higher academic self‐efficacy and a stronger intention to engage ethically with the virtual learning environment. This reduces psychological distancing and enhances perceived accountability, thereby fostering adherence to ethical norms. Conversely, negative attitudes may weaken the perceived ‘academic contract,’ increasing the likelihood of disengagement and misconduct.

The current study found that students with more positive attitudes toward e‐learning showed significantly higher adherence to ethical principles in virtual education, although the observed effect size was small (r = 0.168, β = 0.167). Although statistically significant, this represents a weak association by conventional benchmarks (e.g., Cohen's guidelines), suggesting that, although a meaningful relationship exists, its practical magnitude in isolation is limited. This aligns with previous research indicating that favorable views of online learning are connected to greater academic integrity and ethical awareness [2, 7]. The statistically significant association, even if modest in magnitude, suggests that, in resource‐limited settings—where institutional and infrastructural supports are often lacking—students' perceptions of the e‐learning environment itself can be a meaningful, modifiable factor influencing ethical engagement. Specifically, students who regarded e‐learning as an effective educational tool and viewed instructors as active facilitators were more likely to report ethical behavior. It is important to note that the regression model accounted for only a limited portion of the variance in ethical practice (R^2^ = 0.149), underscoring that numerous other individual, pedagogical, and contextual factors not captured in this study undoubtedly play substantial roles. These findings therefore support research emphasizing the role of instructional design and instructor engagement as among the many factors that foster ethical conduct in virtual settings [1, 5, 7]. However, conflicting evidence exists. For example, Almahasees et al. [34] and Adarkwah [17] reported that positive attitudes toward e‐learning do not always lead to ethical behavior, especially in contexts where institutional policies are weak or where students see online environments as opportunities for academic dishonesty. These differences may be due to variations in cultural norms, technological infrastructure, and institutional readiness for virtual education.

It is also crucial to consider the study's context. The implementation of virtual education at Bushehr University of Medical Sciences occurred rapidly during the COVID‐19 pandemic, despite incomplete infrastructure readiness. As a result, students' positive attitudes may have been driven more by necessity than by genuine preference, which could influence their ethical engagement [3, 4].

### 4.2 E‐Learning Environment and the Role of Instructors

The findings highlighted that those two components—perceiving e‐learning as an effective environment and recognizing instructors as learning facilitators—were significantly associated with ethical behavior. This supports prior research suggesting that well‐structured virtual environments and active instructor presence can reduce academic misconduct and promote student responsibility [1, 5, 7]. For instance, King et al. [8] found that limited instructor‐student interaction in online settings contributed to increased cheating, while Bretag et al. [7] emphasised the role of transparent policies and ethical modelling by educators. Nonetheless, some studies have shown that even in supportive virtual environments, ethical challenges persist. Cicha et al. [18] noted that anonymity and lack of physical supervision in online learning can still lead to unethical behavior. Instructors' limited experience with digital tools may also hinder their ability to enforce ethical standards effectively. Despite these concerns, the present study reinforces the importance of investing in instructor training and virtual classroom design to promote ethical engagement.

### 4.3 Student Interest and Academic Performance

The study showed that a strong interest in one's field of study was a significant positive predictor of ethical behavior, while a GPA below 16 was a negative predictor. These findings align with previous research indicating that academic motivation and performance are closely related to ethical conduct [6, 8]. Students who are more engaged and academically successful tend to be more committed to professional standards and less likely to participate in dishonest practices. Conversely, students with lower academic performance may face increased academic pressure or have reduced self‐confidence, which can raise their likelihood of unethical behavior. These findings suggest that academic support programs aimed at low‐performing students could help promote ethical behavior in virtual education.

### 4.4 Perceived Stress

Contrary to expectations and some prior literature [5, 31, 32], perceived stress was not a significant predictor of ethical behavior in this study. This null finding warrants careful interpretation. The use of a global stress measure (PSS‐14) may not capture specific academic or digital learning stressors that could uniquely impact ethical decision‐making. Furthermore, our study did not assess potential buffering factors—such as individual coping mechanisms, resilience, or social support—which might explain the lack of a direct association in this context. While cultural factors or adapted coping during the pandemic are plausible speculative explanations, they remain hypotheses for future research rather than conclusions from our data. Therefore, this result suggests that the relationship between stress and ethical behavior in virtual education may be more complex, contingent, or indirect than previously assumed, necessitating more nuanced investigation.

### 4.5 Practical Implications for Institutional Practice, Training, and Policy

The findings of this study offer several actionable implications for educational stakeholders in resource‐limited settings. For institutional practice, administrators should prioritize investments in reliable digital infrastructure and user‐friendly e‐learning platforms to foster positive student attitudes. Creating clear, accessible codes of ethical conduct specifically tailored for virtual environments is essential. For professional training, curriculum developers should integrate modules on digital ethics and stress management into health sciences programs. Faculty development initiatives must equip instructors with pedagogical strategies for effective online facilitation, which our study identifies as a key driver of ethical behavior. For policymaking, educational authorities should develop guidelines that recognize the unique challenges of virtual education in under‐resourced settings, moving beyond policies designed for stable, well‐funded contexts. These guidelines should promote a balance between technological investment, pedagogical support, and ethical safeguarding.

### 4.6 Strengths, Limitations, and Future Research Directions

#### 4.6.1 Strengths of the Study

This study possesses several methodological strengths. First, it employs validated instruments, including a researcher‐developed scale with robust psychometric properties for measuring ethical practice. Second, the conceptual framework (Figure 1) provided an a priori theoretical guide for hypothesis testing, thereby enhancing the study's explanatory power relative to purely descriptive approaches. Third, focusing on a resource‐limited, non‐Western setting addresses a critical contextual gap in the literature, offering insights often absent from dominant discourses shaped by experiences in technologically advanced environments. Finally, the concurrent examination of attitudinal, psychological, and ethical variables within a single model allows for a more holistic analysis of the virtual learning experience.

### 4.7 Limitations of the Study

Although this study provides valuable insights into the ethical aspects of virtual education, it has several limitations that should be considered when interpreting the results.

First, the cross‐sectional design precludes establishing causality or directional inference. Although regression analysis identifies statistical predictors, it cannot confirm causal relationships. Longitudinal or experimental designs are required to examine whether attitudes toward e‐learning causally influence ethical practice over time.

Second, data collection relied on self‐reported questionnaires, which are naturally susceptible to social desirability bias, particularly in sensitive areas such as ethics and stress. Participants might have overstated their ethical behavior or understated their stress levels to seem better, which could affect the accuracy of the results.

Third, the study was conducted at a single institution — Bushehr University of Medical Sciences — which limits the generalizability of the findings to other settings. Although the choice of this university was intentional to capture experiences in a resource‐limited context, the unique cultural, infrastructural, and educational environment of BPUMS may not be representative of other institutions, even under similar resource constraints. Therefore, the findings should be interpreted with caution, and future multi‐institutional studies across diverse contexts are needed to enhance external validity.

Furthermore, although the key assumptions of parametric tests were met, our study did not conduct an a priori power analysis. The sample size was estimated using a rule‐of‐thumb approach. Although our final sample (*n* = 256) exceeded the minimum requirement and the main association was significant, some analyses, particularly subgroup comparisons, may have been underpowered to detect smaller effects.

Fourth, the measurement of ethical practice relied on a self‐reported, researcher‐developed instrument. Although the questionnaire demonstrated strong psychometric properties (CVI = 0.93, CVR = 0.84, Cronbach's α = 0.92), its objectivity may be questioned, as it had not been used in broader populations. Furthermore, the high mean score on the ethical practice scale (149.74 ± 16.87 out of 175) may reflect social desirability bias, wherein participants overreported ethical behaviors due to the sensitivity of the topic. While we ensured anonymity to mitigate this bias, future studies could benefit from incorporating mixed‐methods approaches, such as observational data or qualitative interviews, to triangulate self‐reported ethical behaviors.

Fifth, while a significant statistical association was found, the regression model explained a limited proportion of the variance in ethical practice (R^2^ = 0.149). This indicates that e‐learning attitudes, though significant, are among the many contributing factors. A substantial portion of the variance is likely attributable to unmeasured variables, such as personality traits (e.g., conscientiousness, moral identity), specific instructional design features, or broader institutional culture, which should be investigated in future research.

Sixth, although the study accounted for several demographic factors, it did not include other potentially influential variables such as personality traits, socioeconomic status, mental health conditions, or social support. These unmeasured factors could have impacted students' stress levels and ethical behavior.

Seventh, the conceptualization and measurement of stress in this study represent a limitation. The use of the Global Perceived Stress Scale (PSS‐14) may have obscured potential relationships between specific stress subtypes relevant to online learning (e.g., academic stress, technostress) and ethical outcomes. Furthermore, key variables that could moderate or mediate the stress‐ethics relationship, such as coping self‐efficacy, resilience, or perceived social support, were not assessed, limiting our ability to fully interpret the non‐significant finding.

Eight, the research was conducted during the COVID‐19 pandemic, a time characterized by rapid and often unplanned shifts to virtual education. The unique circumstances of the pandemic—including increased psychological stress, limited digital literacy, and infrastructure challenges—may have influenced students' experiences, attitudes, and behaviors in ways that might not reflect how they would respond under more stable or post‐pandemic conditions.

Furthermore, while the study context is explicitly framed as resource‐limited based on institutional descriptors (e.g., recent e‐learning infrastructure, pandemic‐driven adoption), we did not include direct, individual‐level measures of resource constraints (e.g., digital access quality, socioeconomic status) in our quantitative model. Thus, the variable ‘resource limitation’ operates as a contextual descriptor rather than a measured construct, which limits our ability to statistically isolate its specific moderating effects.

Finally, the study employed a quantitative approach and did not explore the qualitative aspects of students' experiences with virtual education. Future research employing qualitative or mixed‐methods designs could yield a deeper understanding of the emotional and contextual factors that influence ethical behavior in online learning environments.

### 4.8 Future Research Directions

Building on these limitations, future studies should employ longitudinal designs to establish temporal precedence and explore causal pathways. Research should also aim to develop more comprehensive models by integrating stronger predictors, such as personality constructs, motivational factors, and detailed pedagogical variables. Specifically, with respect to stress, investigations should distinguish among types of stress and include potential moderators (e.g., coping strategies, social support) to clarify its complex relationship with ethical behavior. Mixed‐methods approaches would be valuable for uncovering the nuanced contextual and emotional factors shaping ethics in digital learning environments, particularly across diverse resource settings.

Future studies should operationalize ‘resource limitation’ using measurable indicators (e.g., device type, internet stability, access to a quiet study space, institutional support scores) to enable direct statistical comparisons of how varying degrees of resource constraints moderate the relationships among attitudes, stress, and ethics.

## 5 Conclusion

This study highlights a modest but significant association between students' attitudes toward e‐learning and their ethical behavior in virtual education settings. A more positive view of e‐learning — especially when students perceive it as an effective learning tool and recognize instructors as active facilitators — was associated with greater adherence to ethical principles. In contrast, perceived stress did not significantly predict ethical behavior, indicating that other contextual or individual factors may have a greater impact on students' ethical decision‐making. While the independent explanatory power of e‐learning attitudes is limited, fostering a positive perception of the virtual learning environment can be one viable component of a broader institutional strategy to promote academic integrity and student well‐being.

Additionally, academic performance and intrinsic interest in one's field of study were key factors influencing ethical engagement. Students with lower GPAs and less interest in their academic discipline tended to report lower levels of ethical conduct, highlighting the need for targeted academic and motivational support.

These results have important implications for educational policymakers and university administrators. Efforts to promote ethical behavior in virtual learning should focus not only on fostering positive attitudes toward e‐learning but also on developing supportive digital infrastructures, instructor training, and student‐centered learning environments. Moreover, integrating ethics education and stress management into the curriculum can further improve students' academic integrity and mental health.

Future research should adopt longitudinal and mixed‐methods designs to establish causality and explore the contextual nuances shaping these relationships, particularly in under‐resourced settings. Comparative studies across diverse contexts and investigations into targeted educational interventions will be vital for developing equitable and ethical virtual education models in global health sciences.

## Author Contributions

**Mohammad Amin Shadman:** investigation, writing – original draft. **Farahnaz Kamali:** methodology, supervision. **Razieh Bagherzadeh:** methodology, writing – review and editing, formal analysis, supervision, validation, data curation. **Shahnaz Pouladi:** conceptualization, writing – original draft, methodology, writing – review and editing, project administration, supervision, validation, visualization, software, data curation.

## Ethics Statement

The research has an ethics code number of IR. BPUMS. REC.1401.084 from the Research Vice‐Chancellor of Bushehr University of Medical Sciences. All study participants provided written informed consent.

## Conflicts of Interest

The authors declare no conflicts of interest.

## Transparency Statement

Shahnaz Pouladi affirms that this manuscript is an honest, accurate, and transparent account of the study being reported; that no important aspects of the study have been omitted; and that any discrepancies from the study as planned have been explained.

## Data Availability

The data that support the findings of this study are available on request from the corresponding author. The data are not publicly available due to privacy or ethical restrictions. The datasets used and/or analyzed during the current study are available from the corresponding author upon reasonable request.
